# Health related quality of life utility weights for economic evaluation through different stages of chronic kidney disease: a systematic literature review

**DOI:** 10.1186/s12955-020-01559-x

**Published:** 2020-09-21

**Authors:** Jacie T. Cooper, Andrew Lloyd, Juan Jose Garcia Sanchez, Elisabeth Sörstadius, Andrew Briggs, Phil McFarlane

**Affiliations:** 1 Avalon Health Economics, Morristown, NJ USA; 2 Acaster Lloyd Consulting Ltd., London, UK; 3 Health Economics and Payer Evidence Lead, Global Market Access & Pricing, AstraZeneca, Academy House, 136 Hills Road, Cambridge, CB2 8PA UK; 4 Price and Market Access Director, Global Market Access & Pricing. AstraZeneca, Mölndal, Sweden; 5 Department of Health Services Research & Policy, London School of Hygiene & Tropical Medicine, London, UK; 6 University of Toronto, Toronto, Canada

**Keywords:** EQ-5D, SF-6D, HUI, Cost-utility, Cost-effectiveness, Dialysis, Transplant

## Abstract

**Background:**

A Task Force from the International Society of Pharmacoeconomics and Outcomes Research (ISPOR) provides recommendations on how to systematically identify and appraise health state utility (HSU) weights for cost-effectiveness analyses. We applied these recommendations to conduct a systematic review (SR) to identify HSU weights for different stages of chronic kidney disease (CKD), renal replacement therapy (RRT) and complications.

**Methods:**

MEDLINE® and Embase were searched for interventional and non-interventional studies reporting HSU weights for patients with CKD stages 1–5 or RRT. As per ISPOR Task Force Guidance, study quality criteria, applicability for Health Technology Assessment (HTA) and generalisability to a broad CKD population were used to grade studies as either 1 (recommended), 2 (to be considered if there are no data from grade 1 studies) or 3 (not recommended).

**Results:**

A total of 17 grade 1 studies were included in this SR with 51 to 1767 participants, conducted in the UK, USA, Canada, China, Spain, and multiple-countries. Health related quality of life (HRQL) instruments used in the studies included were EQ-5D-3L (10 studies), SF-6D (4 studies), HUI2/HUI3 (1 study), and combinations (2 studies). Although absolute values for HSU weights varied among instruments, HSU weights decreased with CKD severity in a consistent manner across all instruments.

**Conclusions:**

This SR identified HSU weights for a range of CKD states and showed that HRQL decreases with CKD progression. Data were available to inform cost-effectiveness analysis in CKD in a number of geographies using instruments acceptable by HTA agencies.

## Background

Chronic kidney disease (CKD) has a substantial impact on patients’ health and life expectancy. CKD has been estimated to affect between 10 and 15% of the population in the U.S. and Canada [1, 2]. CKD can be a progressive disease and the leading causes include diabetes (38%), high blood pressure (26%), and glomerulonephritis (16%) [3]. Progression to end-stage renal disease (ESRD) leaves the patients reliant on dialysis or a kidney transplant [4]. CKD also leads to substantial healthcare resource use. The total Medicare spending on both CKD and ESRD was over $114 billion in 2016 [5].

The KDIGO (Kidney Disease: Improving Global Outcomes) 2012 guidelines recommended that CKD patients should be categorised based on cause, glomerular filtration rate (GFR) category, and albuminuria category in order to aid in predicting CKD prognosis.

Despite guideline directed management of risk factors and use of renin angiotensin aldosterone system inhibitors (RAASi), disease progression, adverse clinical outcomes and mortality rates remain high in patients with CKD, particularly in those patients at risk such as those with moderately or severely increased albuminuria, highlighting a clinical need for new treatments to delay renal disease progression and improve health related quality of life (HRQL).

Since the introduction of health technology assessment (HTA) agencies across the world, the decision to adopt new treatments is becoming more frequently based on the results of cost-effectiveness analyses. The cost-effectiveness of new treatments is influenced by HRQL weights (referred to as health state utility [HSU] weights). HSU weights range between 0 and 1, with 1 representing the valuation of perfect health and 0 representing the valuation of death and are used to estimate quality adjusted life years (QALYs). A systematic review (SR) reported that most cost-effectiveness models in CKD utilised a framework based on disease progression defined by a worsening in GFR stage or albuminuria category [6].

**Fig. 1:**
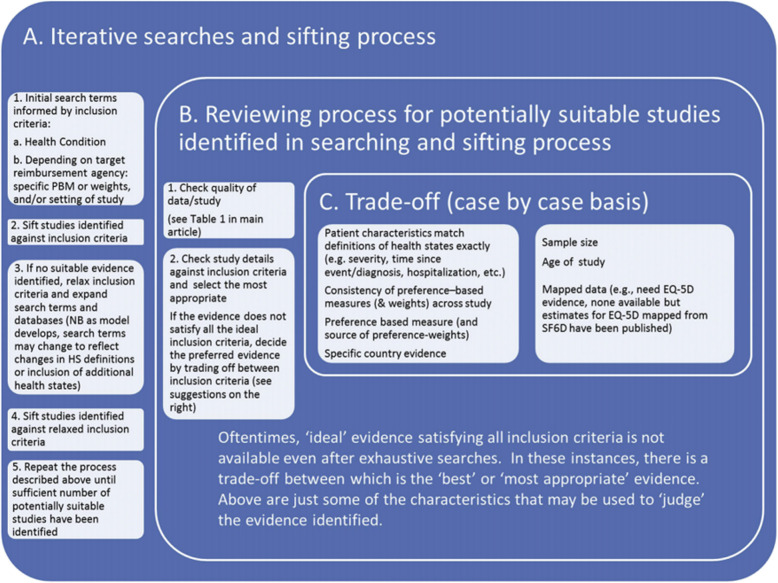
Brazier (2019) HSU identification and selection process. Abbreviations: HSU, health state utility

A Task Force from the International Society of Pharmacoeconomics and Outcomes Research (ISPOR) led by Brazier and colleagues (2019) provided recommendations on how to systematically identify and appraise HSU weights for cost-effectiveness analysis. The recommendations were divided into four sections which describe: (1) iterative search strategy; (2) review process to include studies based on inclusion criteria and data quality; (3) data to be extracted from each study; (4) basis for selecting HSUs to inform a cost-effectiveness analysis (e.g. data availability for a country of interest or data availability using a specific instrument) (Fig. 1).

The impact of dialysis and renal transplantation on HSU weights has been reported in previous SRs, however, it remains uncertain how the magnitude of HSU weights change as CKD progresses between stages 1 and 5 [7–11].

The aim of this SR was to identify HSU weights to inform cost-effectiveness modelling in CKD applying current best practices, and the review was conducted to provide an international perspective.

## Methods

### Search strategy

This SR was based on a prespecified protocol and conducted in accordance with the standards prescribed by the ISPOR Task Force (but also reflects best practice at the Centre for Reviews and Dissemination, National Institute for Health and Care Excellence (NICE), and the Cochrane Collaboration) [12–15]. The search was conducted in both MEDLINE (PubMed) and Embase (OVID) in August 2019. The full search strategy is provided in Additional file 1. Grey literature searches included conference proceedings of three major nephrology congresses and one health economics congress held between 2017 and 2019, and reports from four major HTA agencies (Additional file 2). The bibliographies of relevant published SRs and cost-effectiveness analyses were hand-searched to find additional articles that were not identified in the electronic database searches.

Two independent reviewers (JC, JGS) screened the title and abstract of each record (stage 1), as well as the full texts of all potentially eligible records identified in stage 1 (stage 2). A third independent reviewer (AL) resolved any disagreements.

Study inclusion criteria are shown in Table 1 and are based on the PICOS (population, intervention, comparator, outcome, study) framework.

**Table 1:** Criteria for including studies in the review

i. Population	*People with any stage CKD including patients on dialysis (haemodialysis or peritoneal dialysis) or with a renal transplant; any gender, any location, and any severity of CKD. Populations must be representative of the CKD population (*i.e.*, general comorbidities, reasonable age range) and be greater than 25 people in size. Subgroups of interest include (not limited to): CKD patients with albuminuria (normo-, micro-, macro-albuminuria), T2DM, glomerulonephritis, IgA nephropathy.*
ii. Interventions/comparators	*All interventions and comparative data were included. Where the intervention is not relevant for the study purposes in some cases only baseline or placebo arm data is included.*
iii. Outcomes	*Health state utilities from standardised generic multi-attribute utility measures such as EQ-5D, SF-6D or Health Utilities Index (HUI).* *HSUs for all CKD stages, dialysis modalities (haemodialysis and peritoneal dialysis), or renal transplant.* *Disutility associated with cardiovascular events commonly included in health economic models in CKD (acute and chronic where available): myocardial infarction, stroke, heart failure.* *Disutility associated with adverse events commonly included in health economic models in CKD: potassium imbalances (hypo- and hyperkalaemia), volume depletion, acute kidney injury, major hypoglycaemic events, diabetic ketoacidosis, fractures, amputations (minor/major or toe, foot, limb,* etc.*).* *Impact of comorbidities or patient characteristics on HSUs: albuminuria (normo-, micro-, macro-albuminuria), T2DM, hypertension, heart failure or cardiovascular disease, age or sex on HSUs.* *Impact of complications related to renal replacement therapies on HSUs: dialysis related complications (*e.g. *vascular access thrombosis), renal transplant failure, renal transplant surgery.*
iv. Study Designs	*Interventional or non-interventional research.*
v. Other requirements	*Records from January 1, 1999 to present (August, 2019) only.* *Abstract and full-text must be available in English text.*

Abbreviations: *CKD* chronic kidney disease, *EU5* France, Germany, Italy, Spain, UK, *HSU* health state utility, *HUI* health utility index, *IgA* immunoglobulin *A* SF-6D, Short Form questionnaire-6 Dimensions, *T2DM* type 2 diabetes

### Critical appraisal

Each study was assessed against the following criteria:

- The study was conducted in a CKD population
- The study reports original empirical HSU weights
- Data were collected using a generic HRQL measure (i.e. EQ-5D, short-form 6-dimention [SF-6D] or a mappable equivalent such as short-form 36 [SF-36] or short-form 12 [SF-12]; or the Health Utility Index [HUI])
- The study sample size was at least 25 patients
- The study was conducted in a country of interest (i.e., USA, Canada, Australia, China, UK, Spain, Italy, France or Germany)
- HSU weights were presented in a comprehensive way that is useful to inform cost-effectiveness analysis (e.g. HSU weights were available by CKD stage)

To weigh both data quality and data appropriateness as recommended by Brazier and colleagues (2019), each study that met the critical appraisal at stage 1 was then reviewed in full in stage 2 and graded from 1 to 3 with consideration to the presence of bias, alignment with HTA criteria, and general compliance with our initial selection criteria (Table 2). To assess bias, each study’s methodology was examined for selection bias, bias in data analysis or interpretation, drop out or missing data, or bias in study execution such as unblinding in randomised control trials.

**Table 2:** Record Grading Scale

1	Study meets all HTA selection criteria and has no apparent sources of significant bias
2	Study meets HTA selection criteria but may be subject to bias (e.g. may need the application of a mapping algorithm to derive HSU weights or there may be study methodology bias)
3	Study does not meet HTA selection criteria (e.g. not a population representative of the CKD population)

Abbreviations: *CKD* chronic kidney disease, *HTA* health technology assessment, *HSU *health state utility

Grade 1 studies were considered most appropriate for HTA. If data for a specific health state was not available using Grade 1 studies, then, Grade 2 studies would be reviewed to identify a missing value following the iterative approach recommended by Brazier and colleagues (2019). Grade 3 studies were considered to be inappropriate.

Relevant data, as recommended by Brazier and colleagues (2019), was extracted from the included studies into a prespecified extraction grid.

## Results

**Fig. 2:**
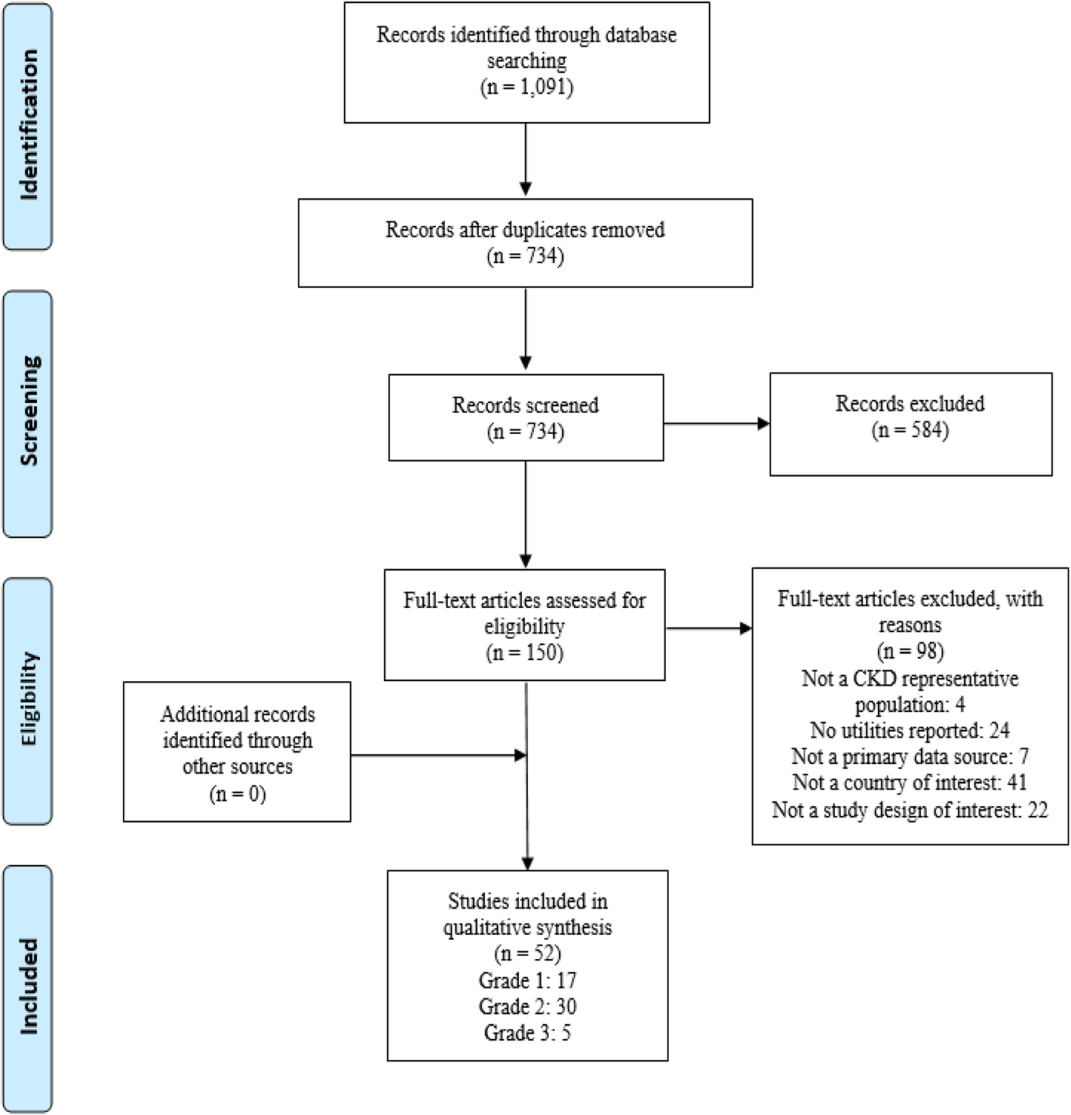
PRISMA Diagram. Abbreviations: CKD, chronic kidney disease; PRISMA, Preferred Reporting Items for Systematic Reviews and Meta-Analyses

Electronic database searches identified 1091 records. After title/abstract screening, 150 studies were selected for full-text review, of which 52 met the final inclusion criteria. The grey literature identified 83 studies, although no new studies met our inclusion criteria (Additional file 2). The article selection process is displayed in Fig. 2.

Of the 52 included studies, the grading process identified 17 Grade 1, 30 Grade 2, and 5 Grade 3 studies (Additional file 3). Data were extracted for the Grade 1 studies (Additional file 4). Fourteen of the studies reported more than one CKD HSU weight, resulting in a total of 58 CKD HRQL estimates across different health states (i.e., CKD stages, haemodialysis (HD), peritoneal dialysis (PD) and renal transplant (Trx)).

Ten studies (59%) used the EQ-5D-3L, four (24%) used the SF-6D, one (6%) used the HUI3, and two (12%) used multiple instruments (HUI2 and HUI3; EQ-5D-3L and HUI3). Of the reported HSU weights, 18 (41%) described dialysis patients, 17 (39%) described transplant patients, and 9 (21%) described cohorts by CKD stages. Studies were reported from Canada (*n* = 4; 29%), the UK (*n* = 3; 18%), the US (*n* = 3; 28%), Spain (*n* = 2; 12%), and China (*n* = 2; 12%), and two studies (12%) were multinational. A summary of key study characteristics is reported in Table 3.

**Table 3:** Main study characteristics

Author (Year)	N Mean age (SD)	Males	Study Type	Setting and location	Population overview
Blakeman (2014)	221 patients. 71.8 (9.0) years.	41.2%	Randomised controlled trial.	General practice. Greater Manchester, UK.	Stage 3 CKD only.
Briggs (2016)	3547 patients. 54.3 (14.3) years.	59.9%	Randomised controlled trial.	500 dialysis centres. 22 countries.	Moderate to severe secondary hyperparathyroidism on HD.
Davison (2008)	185 patients. 63.6 (12.2) years.	55.0%	Prospective observational study.	10 dialysis/ renal insufficiency units. Alberta, Canada.	CKD stage 4 and 5 expected to start dialysis within 12 months. Patients currently receiving HD or PD (started in last 12 months).
Davison (2009)	185 patients. 63.6 (12.2) years.	55.0%	Prospective observational study.	10 dialysis/ renal insufficiency units. Alberta, Canada.	CKD stage 4 and 5 expected to start dialysis within 12 months. Patients currently receiving HD or PD (started in last 12 months).
Gorodetskaya (2005)	271 patients. 62.8 (12.7) years.	48%	Prospective observational study.	Patients from a single nephrology and dialysis site. USA.	Two groups defined by: -GFR between 30 and 70 ml/min. -GFR < 30 ml/min.
Jardine (2017)	200 patients. 51.8 (12.1) years.	69.5%	Randomised controlled trial.	40 home and hospital dialysis centres. Australia, China, Canada, and New Zealand.	Adult patients requiring maintenance HD.
Jesky (2016)	745 patients. 64, 95% CI (50–76) years.	60.8%	Prospective observational study.	Two large hospitals. Birmingham, UK.	Pre-dialysis CKD and GFR < 30 ml/min.
Lee (2005)	416 patients. Males: 58.2 years. Females: 55.5 years.	58.9%	Cross-sectional study.	Renal unit departmental database. South Wales, UK.	Patients receiving HD, waiting to start HD or after receiving a renal transplant.
Manns (2002)	128 patients. 61.8, 95%CI (59.1, 64.6) years.	56.3%	Cross-sectional study.	Southern Alberta Renal Program. Alberta, Canada.	All participants had received over 6 months of HD.
Manns (2003)	192 patients. 60.8, 95%CI (58.6, 63.0) years.	55.7%	Cross-sectional study.	Southern Alberta Renal Program. Alberta, Canada.	All participants had received over 6 months of HD.
Manns (2009)	51 patients. 54.1 years.	62.5%	Randomised controlled trial.	Southern Alberta Renal Program. Alberta, Canada.	In- or home conventional HD 3 times weekly.
Neri (2011)	386 patients. GFR > 90 ml/min = 48.1 (16.2) years. GFR 90–60 ml/min = 52.2 (13.4) years. GFR 59–30 ml/min = 51.5 (11.8) years. GFR 29–15 ml/min = 52.2 (12.1) years. GFR < 15 ml/min = 43.2 (14.6) years.	61.4%	Cross-sectional study.	Two outpatient clinics. Midwest, USA.	Kidney transplant patients.
Ortega (2007)	307 patients. 51.6 (12) years.	59.2%	Prospective observational study.	16 hospitals. Spain.	Adult patients with end-stage renal disease who received a kidney transplant.
Ortega (2009)	162 patients. 55.8 (12.3) years.	NR	Prospective observational study.	Four hospitals. California, USA.	Pre-renal transplant and 12 months post-renal transplant.
Ortega (2013)	206 patients. 53.4 (12.9) years.	61.2%	Cross-sectional study.	39 transplantation units. Spain.	Renal transplant patients 6–24 months post-renal transplant.
Pan (2018)	315 patients. 57.3 (14.9) years.	54.9%	Cross-sectional study.	First Affiliated Hospital of Soochow University. Eastern China.	Patients on HD.
Wong (2019)a	399 patients. 57.3 (12.7) years.	61.9%	Cross-sectional study.	Hospital, community HD centres or home HD or PD. Hong Kong.	Patients undergoing home based nocturnal HD, PD, hospital or community HD.

Abbreviations: *CKD* chronic kidney disease, *GFR* glomerular filtration rate, *HD* haemodialysis, *PD* peritoneal dialysis, *SD* standard deviation

HSU weights for the different CKD health states are reported in Table 4**.** HRQLs for haemodialysis and post-transplant patients were the most common. There is a scarcity of data describing HRQL for patients in CKD stages 1–5; only one study was identified that reported an HSU value for stage 2 patients and no studies reported HSUs for stage 1 patients.

**Table 4:** HSU weights by subgroup

	Utility Value (SD)	Sample Size (N)	Instrument	Country	Source
**CKD Stage 2**					
	0.85 (95% CI: 0.70–1)	29	EQ-5D-3L	UK	[16]
**CKD Stage 3**					
Stage 3a	0.80 (95% CI: 0.69–1)	45	EQ-5D-3L	UK	[16]
Stage 3b	0.80 (95% CI: 0.68–1)	173	EQ-5D-3L	UK	[16]
Stage 3	0.67 (0.31)	50	HUI3	USA	[17]
**CKD Stage 4**	0.74 (95% CI: 0.62–0.85)	423	EQ-5D-3L	USA	[16]
	0.55 (0.34)	65	HUI3	USA	[17]
**CKD Stage 5**	0.54 (0.36)	28	HUI3	USA	[17]
	0.73 (95% CI: 0.62–1)	75	EQ-5D-3L	UK	[16]
**Haemodialysis**	0.75 (0.25)	1767	EQ-5D-3L	Various	[18]
	0.44 (0.32)	99	EQ-5D-3L	UK	[19]
	0.78 (0.24)	200	EQ-5D-3L	Various	[20]
	0.60 (95% CI: 0.55, 0.64)	128	EQ-5D-3L	Canada	[21]
	0.69 (95% CI: 0.63,0.76)	51	EQ-5D-3L	Canada	[22]
	0.54 (0.31)	271	HUI3	USA	[17]
	0.75 (0.11)	315	SF-6D	China	[23]
	0.73 (0.11)	135	SF-6D	China	[24]
	0.78 (0.09)	41	SF-6D	China	[24]
	0.79 (0.11)	118	SF-6D	China	[24]
**Peritoneal Dialysis**	0.53 (0.34)	64	EQ-5D-3L	UK	[19]
	0.78 (0.11)	103	SF-6D	China	[24]
**Unspecified Dialysis**	0.54 (0.31)	38	HUI3	USA	[17]
	0.74 (0.20)	185	HUI2	Canada	[25]
	0.58 (0.26)	185	HUI3	Canada	[25]
	0.67 (0.13)	185	SF-6D	Canada	[26]
**Pre-Transplant**	NR				
**Post-Transplant**					
CKD stage 1–2	0.79 (0.25)	386	HUI3	USA	[27]
CKD stage 3	0.87 (0.14)	172	EQ-5D-3L	USA	[27]
CKD stage 3	0.75 (0.26)	172	HUI3	USA	[27]
CKD stage 4	0.87 (0.10)	51	EQ-5D-3L	USA	[27]
CKD stage 4	0.74 (0.22)	51	HUI3	USA	[27]
CKD stage 5	0.82 (0.12)	19	EQ-5D-3L	USA	[27]
CKD stage 5	0.67 (0.33)	19	HUI3	USA	[27]
CKD stage not reported	0.71 (0.27)	209	EQ-5D-3L	UK	[19]
CKD stage not reported	0.77 (NR)	126	SF-6D	Spain	[28]
CKD stage not reported	0.76 (NR)	80	SF-6D	Spain	[28]

Abbreviations: *CKD* chronic kidney disease, *HUI2* Health Utilities Index Mark 2, *HUI3* Health Utilities Index Mark 3, *NR* not reported, *RRT* renal replacement therapy, *SD* standard deviation, *SF-6D* Short Form questionnaire-6 Dimensions

Four longitudinal studies reported HSU weights. Limited data were available describing HRQL changes with disease progression. Regarding HRQL in patients undergoing RRT, HSU weights increased with time (Table 5).

**Table 5:** HSU weights reported in longitudinal studies

	Baseline	3 Months	6 Months	12 months	Country	Instrument	Source
**CKD stage 2**	NR						
**CKD stage 3**	0.67 (0.30)		0.67 (0.29)		UK	EQ-5D-3L	[29]
**CKD stage 3a**	NR						
**CKD stage 3b**	NR						
**CKD stage 4**	NR						
**CKD stage 5**	NR						
**Haemodialysis**	0.65 (0.027)			0.62 (0.030)	Canada	EQ-5D-3L	[30]
**Peritoneal dialysis**	0.64 (0.063)			0.67 (0.046)	Canada	EQ-5D-3L	[30]
**Transplant**	**Pre-Transplant**	**Post-Transplant**					
	0.74 (0.21)	0.81 (0.19)	1.0 (0)	0.82 (0.20)	Spain	EQ-5D-3L	[31]
	0.61 (NR)			0.74 (NR)	USA	EQ-5D-3L	[32]
	0.78 (NR)			0.86 (NR)	USA	EQ-5D-3L	[32]

Abbreviations: *CKD* chronic kidney disease, *HSU* health state utility, *NR* not reported

**Fig. 3:**
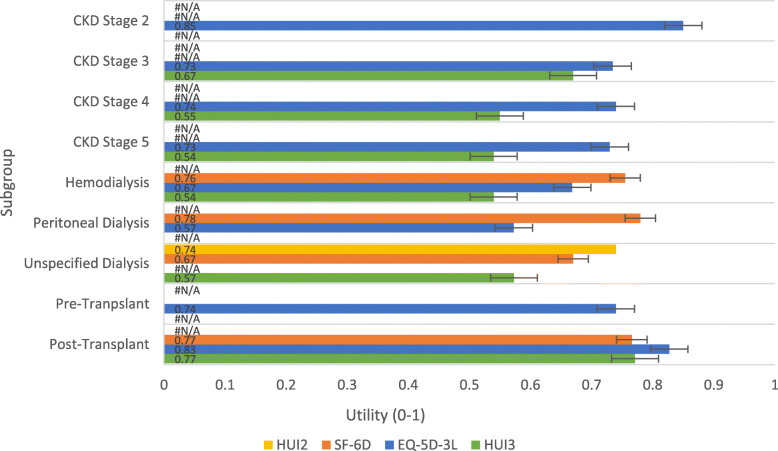
Mean HSU weights by state presented by instrument. HSU values are weighted averages calculated using subgroup population sizes; Error bars represent standard error. Abbreviations: CKD, chronic kidney disease; HSU, health state utility; HUI2, Health Utilities Index Mark 2; HUI3, Health Utilities Index Mark 3; SF-6D, Short Form questionnaire-6 Dimensions

Mean weighted HSU weights for the different CKD health states according to instrument are reported in Fig. 3. There is clear variation in utility values across instruments. However, there is an overall consistent trend with each instrument showing a reduction in HRQL with CKD progression. HRQL is lowest with dialysis. HSU weights reported using SF-6D indicate no difference between haemodialysis and peritoneal dialysis while HSU weights are lower for peritoneal dialysis when using EQ-5D-3L. HRQL increases after renal transplantation.

Only one study identified in our SR reported the impact of adverse events or complications on CKD patients on dialysis [18]. The HSU weights reported are shown in Table 6. No studies reported the impact of adverse events or complications on patients with CKD stage 1–5 or after a renal transplant.

**Table 6:** HSU weights related to complications in patients on haemodialysis [18]

Complications	EQ-5D-3L Score (95% Confidence Interval)	
	Acute Effect (week 1–13)	Chronic Effect (week 14–244)
Myocardial Infarction	0.52 (0.47–0.58)	0.66 (0.57–0.76)
Hospitalisation for unstable angina	0.54 (0.46–0.63)	0.60 (0.49–0.71)
Stroke	0.50 (0.41–0.60)	0.49 (0.30–0.68)
Heart Failure	0.58 (0.54–0.63)	0.66 (0.59–0.73)
Bone Fracture	0.35 (0.30–0.40)	0.58 (0.51–0.65)

Abbreviations: *CKD* chronic kidney disease, *HSU* health state utility

Study quality is reported in Additional file 5. Since all analysed studies met our grade 1 screening requirements, overall study quality was high. Quality assessment reported a lack of clarity in 7 studies regarding drop out or missing data rates. Lee et al. reported a low 33% response rate but was retained due to the questionnaire administration method (survey packets were mailed to patients’ houses) [19].

## Discussion

This SR was designed to identify HSU weights for a range of CKD health states using methods promoted by Brazier and colleagues (2019) and other best guidance available. To our understanding, this is the first SR to report HSU weights for CKD stages 2–5, as well as RRT, as previous SRs focused on RRT only [7, 10, 33–37].

The review identified a large number of published studies that reported HSU weights for CKD populations. By focusing to the most generalisable and reliable Grade 1 studies, we hope to present the most accurate summary of HSU weights in CKD.

This is also the first SR to have been undertaken in the area of HSU weights since the Brazier and colleagues (2019) guidance on SR methods for the identification of HSU weights for cost-effectiveness analysis was released [12]. Based on our experience of implementing the guidance, we found that the recommended approach worked well and the guidance provided a very good rationale and set of methods for identifying the most relevant data.

According to the ISPOR Task Force on Indirect Treatment Comparisons Good Research Practices, conducting a meta-analysis on this topic may have been appropriate. However, the Brazier and colleagues (2019) guidance for identification of HSU weights in particular does not specify the need for this type of analysis. We believe this SR more directly addresses the needs of decision-making entities in different countries, as ideal data for decision-making would be country-specific with a relevant presence of comorbidities, settings, HSU instruments, and date-of-publication ranges - entities that may be lost in meta-analysis.

The review found an overall trend across studies for a decline in HSU scores as CKD deteriorated, (based on GFR). This fits with clinical expectation, but it is a point worth making because we believe that it provides some justification or validation of the identified HSU weights. Different factors may affect HRQL decline at different CKD stages. For instance, reductions in HRQL in early CKD stages may be driven by the presence of comorbidities such as diabetes, while a decline in HRQL in more advanced CKD stages may also be driven by an increase in the incidence of heart failure, and cardiovascular complications such as myocardial infarction or stroke which could also have a substantial impact on HRQL [38–41]. However, it is difficult to determine the cause of any decline in HRQL when exploring published data because we are limited to the data that have been included in the publication. This is one important limitation of the published data and of this SR. The studies included varied in terms of their design (cross-sectional survey, randomised trial, prospective observational study) and used different instruments which made comparisons between them challenging. Although a similar declining trend was observed with CKD progression across instruments, absolute HSU values were different across instruments with EQ-5D-3L reporting the highest values. This could reflect that sensitivity to capture the impact of CKD progression on HRQL may be different between the instruments reported in this systematic review. This could also present a challenge when estimating QALYs gained in cost-effectiveness analyses of new treatments for CKD. As a consequence, incremental cost-effectiveness ratios could be different depending on the instrument used and, potentially, this could result in different recommendations for the adoption of new treatments for CKD by HTA agencies. Variability in HSU weights between instruments remains a source of bias when combining results from studies using different instruments. This could be avoided by only including studies which use one specific instrument. While the number of patients assessed differed substantially between studies, by HSU instruments used and CKD stages, this did not seem to influence HSUs reported as they seemed aligned for each instrument and CKD stage regardless of sample size. While HSU weights were lowest for dialysis, it was not clear if HSU differs between haemodialysis and peritoneal dialysis as different trends were noted between the instruments used. Further research should be conducted to increased the understanding of these differences.

A number of aspects may affect the mix of patients included in the studies reported in this SR and, therefore, the eventual HSU weights reported. For example, patients receiving in-centre dialysis may have more comorbidities and complications than patients that are good candidates for peritoneal dialysis, dialysis at home or nocturnal dialysis. Patients with less severe kidney disease or higher HRQL may also be more likely to respond to voluntary questionnaires or participate in trials, potentially skewing the data. Further, it is possible that geographical variations may arise due to differences in clinical practice but also how people interpret HRQL questionnaires.

Regression methods such as those applied by Briggs and colleagues (2012) provide a way to estimate HSUs from longitudinal studies for different CKD stages improving the precision of the effects and understand their origin. Regression analyses of large datasets allow us to understand the impact of CKD related events on HRQL as well as understanding the influence of covariates and so this offers advantages over SR methods. It may also be possible to explore some of these issues with meta-regression type techniques. However, the studies are not consistent in the information that they present which makes it difficult to compare these variables systematically. Alternatively, it could be assumed that a ‘true’ score for a specific health state lies within the range of scores that have been identified from the review for a specific health state. Therefore, cost-effectiveness analysis could be informed by the range of scores as opposed to a single point estimate.

All studies used generic instruments of health rather than disease-specific instruments, but despite this the HSU weight varied substantially between different instruments (Fig. 3). This figure showed that the HUI3 questionnaire produces lower HRQL scores in comparison to the SF-6D and EQ-5D-3L. Higher HSU weights were reported with the SF-6D. SF-6D values for dialysis patients in particular, (0.76, and 0.78 for haemodialysis and peritoneal dialysis, respectively) seemed high considering that these applied to patients receiving dialysis. If the measures are not in agreement, then this could be explored (and perhaps controlled for) using a meta-regression approach. Where possible, HSU weights used in a cost-effectiveness analysis could be limited to a single instrument relevant to the specific research question for a cost-effectiveness analysis such as the EQ-5D-3L for cost-effectiveness analyses submitted to NICE in England.

In 2012, KDIGO provided guidelines for the categorisation of patients according to GFR and albuminuria [42]. Our SR did not find any studies that reported HSU weights based on both GFR and albuminuria or albuminuria alone. A data gap exists to understand the impact of albuminuria on HRQL. Additional data gaps exist around the reporting of HSUs related to CKD stage 1, adverse events and complications in patients with CKD.

## Conclusions

To our knowledge, this is the first SR examining HSU scores for patients with CKD stages 1–5 with stratification by CKD stage. This is also one of the first reviews to apply the Brazier and colleagues (2019) guidance. There were sufficient data to provide weighted mean HSU weights for most CKD stages of interest [2–5] and RRT. No data was found reporting HSUs weights according to the KDIGO 2012 GFR/albuminuria categories. The findings from the SR illustrate how HRQL is worse for patients with worse renal function. Although similar trends were seen, notable differences in absolute values were identified across instruments highlighting potential differences in sensitivity to capture changes in HRQL in patients with CKD. This could result in the estimation of different QALYs gained in cost-effectiveness models and could affect the recommendation to adopt new treatments for CKD by HTA agencies. Regression methods are an option to provide refined HSU values from longitudinal studies while meta-analytical methods could help explore differences when using aggregate data.

## Supplementary information

**Additional file 1.** Search Strategy. PubMed search results.

**Additional file 2.** Grey Literature Search. Grey literature search results.

**Additional file 3.** Full Text Screening. Full text screening results with decision, HTA compatibility, bias assessment, and grade.

**Additional file 4.** HSU weights from studies identified in SLR. Author and year, HRQOL Elicitation and scoring methods, subgroups, and mean utility scores for each grade one study identified in the SLR.

**Additional file 5.** Quality Assessment. Bias assessment using a traffic light grading system of grade one studies identified in SLR.

## Data Availability

Not applicable.

## Abbreviations

CKD: Chronic kidney disease
ESRD: End-stage renal disease
GFR: Glomerular filtration rate
HD: Haemodialysis
HRQL: Health related quality of life
HSU: Health state utility
HTA: Health technology assessment
HUI: Health utility index
HUI2: Health Utilities Index Mark 2
HUI3: Health Utilities Index Mark 3
IgA: Immunoglobulin A
ISPOR: International Society of Pharmacoeconomics and Outcomes Research
KDIGO: Kidney Disease: Improving Global Outcomes
NICE: National Institute for Health and Care Excellence
NR: Not reported
PD: Peritoneal dialysis
QALY: Quality adjusted life year
RAASi: Renin angiotensin aldosterone system inhibitors
RRT: Renal replacement therapy
SD: Standard deviation
SF-12: Short-form 12
SF-36: Short-form 36
SF-6D: Short-form questionnaire-6 dimensions
SR: Systematic review
T2DM: Type 2 diabetes
Trx: Renal transplant
