# Supplementary material for: Health related quality of life utility weights for economic evaluation through different stages of chronic kidney disease: a systematic literature review
Source: Health Qual Life Outcomes. 2020 Sep 21;18:310. doi: 10.1186/s12955-020-01559-x (PMC7507735; doi:10.1186/s12955-020-01559-x)
Supplement: Supplementary file 1 — Additional file 1. Search Strategy. PubMed search results. [file 12955_2020_1559_MOESM1_ESM.docx]

Supplemental Materials 1: Search Strategy

| ID | Search | Comment | Hits |
| --- | --- | --- | --- |
| #1 | "renal insufficiency, chronic"[MeSH Terms] | CKD related terms | 107864 |
| #2 | (“chronic kidney disease”[Title/Abstract] or CKD[Title/Abstract]) | CKD related terms | 42916 |
| #3 | RENAL REPLACEMENT THERAPY[MeSH Terms] | CKD related terms | 196937 |
| #4 | *DIALYSIS[Title/Abstract] | CKD related terms | 103789 |
| #5 | Kidney Transplantation[MeSH Terms] | CKD related terms | 91923 |
| #6 | ("Kidney Transplant*"[Title/Abstract] OR "RENAL Transplant*"[Title/Abstract]) | CKD related terms | 38356 |
| #7 | #1 or #2 or #3 or #4 or #5 or #6 or #7 | CKD related terms | 306303 |
| #8 | "quality adjusted life years"[MeSH Terms] | Utility related terms | 11076 |
| #9 | (("quality adjusted life year*"[Title/Abstract] OR "quality-adjusted life year*"[Title/Abstract] OR QALYs[Title/Abstract] OR QALY[Title/Abstract])) ("eq-5d"[Title/Abstract] OR "EQ 5D"[Title/Abstract] OR "euroqol-5d"[Title/Abstract] OR "euroqol 5d"[Title/Abstract]) | Utility related terms | 18071 |
| #10 | ("Health Utility Index"[Title/Abstract] OR "Health Utilities Index"[Title/Abstract] OR HUI[Title/Abstract]) | Utility related terms | 1649 |
| #11 | ("Short-Form Six-Dimension"[Title/Abstract] OR "Short Form Six Dimension"[Title/Abstract] OR "SF 6D"[Title/Abstract] OR "SF-6D"[Title/Abstract] OR SF6[Title/Abstract]) | Utility related terms | 750 |
| #12 | ("Kidney Disease Quality of Life Instrument"[Title/Abstract] OR "Kidney-Disease Quality of Life Instrument"[Title/Abstract] OR KDQOL[Title/Abstract]) | Utility related terms | 279 |
| #13 | #8 or #9 or #10 or #11 or #12 | Utility related terms | 24761 |
| #14 | #7 and #13 | Total Unfiltered | 735 |
| #15 | #7 and #13 | Total Filtered* | 605 |

* The following filters were applied: human population, publication date from 01/01/1999 to present, and NOT letters, guidelines, case reports
