# Supplementary material for: Health related quality of life utility weights for economic evaluation through different stages of chronic kidney disease: a systematic literature review
Source: Health Qual Life Outcomes. 2020 Sep 21;18:310. doi: 10.1186/s12955-020-01559-x (PMC7507735; doi:10.1186/s12955-020-01559-x)
Supplement: Supplementary file 4 — Additional file 4. HSU weights from studies identified in SLR. Author and year, HRQOL Elicitation and scoring methods, subgroups, and mean utility scores for each grade one study identified in the SLR. [file 12955_2020_1559_MOESM4_ESM.docx]

HSU weights from studies identified in SLR

| **Author (Year)** | **HRQL Elicitation & scoring** | **Groups** | **Utility, Mean (SD) n** |
| --- | --- | --- | --- |
| Blakeman (2014) | EQ-5D  TTO   Assume Dolan UK weights | CKD Stage 3 (Placebo) at baseline  CKD Stage 3 (Placebo) at 6 months | 0.67 (0.30)  0.67 (0.29) |
| Briggs (2016) | EQ-5D-3L  TTO  Dolan UK weights | *Myocardial infarction* Short term effect Long term effect *Hospitalisation for unstable angina* Short term effect Long term effect *Heart failure* Short term effect Long term effect *Peripheral vascular event* Short term effect Long term effect *Stroke* Short term effect Long term effect *Bone fracture* Short term effect Long term effect *Parathyroidectomy* Short term effect Long term effect | Disutilities:  -0.12 -0.10  -0.11 -0.05  -0.11 -0.10  -0.31 -0.20  -0.20 -0.11  -0.31 -0.12  -0.06 0.01 |
| Davison (2008) | HUI-2  Assumed standard HUI methods (VAS & SG)  Canadian public | Pre-dialysis (n=116)  Dialysis (n=62) | 0.78 (0.17)  0.68 (0.22) |
| Davison (2009) | SF-6D  Assume standard SF-6D methods (SG)  Assume UK general public | Pre-dialysis (n=116)  Dialysis (n=62) | 0.69 (0.13)  0.62 (0.13) |
| Gorodetskaya (2005) | HUI-3  Assumed standard HUI-3 methods (VAS & SG)  Assumed Canadian public | GFR>=60 30<=GFR<60 15<=GFR<30 GFR<15, ND GFR<15, dialysis Dialysis  Long term follow up stage 4/5 pts Utility change | 0.67 (0.31) n=22 0.67 (0.31) n=50 0.55 (0.34) n=65 0.54 (0.35) n=28 0.54 (0.33) n=66 0.54 (0.31) n=38   -0.039 per year |
| Jardine (2017) | EQ-5D-3L  NR | Standard haemodialysis: 12-18 hours per week extended hemodialysis:24+ hours per week | 0.76 (0.25) n=100 0.79 (0.23) n=100 |
| Jesky (2016) | EQ-5D-3L  Dolan 1997 UK TTO weights | Stage G1/G2: normal Stage G3a: GFR 45-59 (mild reduction) Stage G3b: GFR 30-44 (moderate/severe) Stage G4: GFR 15-29 (severe reduction) Stage G5: GFR<15 (kidney failure) | 0.85 (0.70, 1.0) n=29 0.80 (0.69, 1.0) n=45 0.80 (0.68, 1.0) n=173 0.74 (0.62, 0.85) n=423 0.73 (0.62, 1.0) n=75 |
| Lee (2005) | EQ-5D-3L  Dolan 1997 UK TTO weights | Peritoneal dialysis Haemodialysis Pre-dialysis Renal Transplant | 0.53 (0.34) n=74 0.44 (0.32) n=95 0.57 (0.33) n=33 0.71 (0.27) n=204 |
| Manns (2002) | EQ-5D-3L  Dolan 1997 UK TTO weights | Kt/V<1.3 Kt/V>=1.3 All patients | 0.45 0.65 0.60 95%CI (0.55, 0.64) |
| Manns (2003) | EQ-5D-3L  Dolan 1997 UK TTO weights | HD Baseline  HD after 12 months  PD baseline PD after 12 months | Mean (std Error) 0.65 (0.027) 0.62 (0.030)  0.64 (0.063) 0.67 (0.046) |
| Manns (2009) | EQ-5D-3L  Dolan 1997 UK TTO weights | Haemodialysis patients, baseline | Mean (95%CI) 0.69 (0.63, 0.76) n=51 |
| Neri (2011) | EQ-5D-3L  Shaw, et al. USA TTO weights | Post-transplant CKD stage 1-2: GFR>60 Post-transplant CKD stage 3: GFR 59-30 Post-transplant CKD stage 4: GFR 29-15 Post-transplant CKD stage 5: GFR <15 | 0.88 (0.15) n=143 0.87 (0.14) n=172 0.87 (0.10) n=51 0.82 (0.12) n=19 |
| Ortega (2007) | EQ-5D-3L  TTO weights, possibly Spanish, report is ambiguous | Pre-transplant (ESRD) 3 months post-transplant 6 months post-transplant 12 months post-transplant | 0.74 (0.21) 0.81 (0.19) 1.0 (0) 0.82 (0.20) |
| Ortega (2009) | EQ-5D-3L  TTO weights, possibly Spanish, report is ambiguous | Pre-transplantation <60yrs Pre-transplantation > 60yrs  Post transplantation <60yrs Post transplantation >60yrs | 0.61 0.78  0.74 0.86 |
| Ortega (2013) | SF-6D  UK standard gamble weights | Graft clinical evaluation: Very good - good Average - bad | 0.78 n=170 0.61 n=24 |
| Pan (2018) | SF-6D   Hong Kong population weights, standard gamble | Total sample | 0.75 (0.11) n=315 |
| Wong (2019)a | SF-6D   Hong Kong population weights, standard gamble | Peritoneal dialysis Hospital haemodialysis Home haemodialysis Community haemodialysis | 0.78 (0.11) n=103 0.73 (0.11) n=135 0.78 (0.09) n=41 0.79 (0.11) n=118 |
| Abbreviations: CKD, chronic kidney disease; ESRD, end-stage renal disease; GFR, glomerular filtration rate; HRQOL, health-related quality of life; HUI, Health Utilities Index; NR, not reported; SD, standard deviation; SF-6D, Short Form questionnaire-6 Dimensions; SG, standard gamble; TTO, time trade-off ; VAS, visual analogue scale | | | |
