# Supplementary material for: Health related quality of life utility weights for economic evaluation through different stages of chronic kidney disease: a systematic literature review
Source: Health Qual Life Outcomes. 2020 Sep 21;18:310. doi: 10.1186/s12955-020-01559-x (PMC7507735; doi:10.1186/s12955-020-01559-x)
Supplement: Supplementary file 5 — Additional file 5. Quality Assessment. Bias assessment using a traffic light grading system of grade one studies identified in SLR. [file 12955_2020_1559_MOESM5_ESM.docx]

| Quality Assessment | **Bias** | | | |
| --- | --- | --- | --- | --- |
|  | Participant selection | Data analysis or interpretation | Drop out or missing data | Study execution |
| **Study** |  |  |  |  |
| Blakeman 2014 | ⬤ | ⬤ | ⬤ | ⬤ |
| Briggs 2016 | ⬤ | ⬤ | ⬤ | ⬤ |
| Davidson 2008 | ⬤ | ⬤ | ⬤ | ⬤ |
| Davidson 2009 | ⬤ | ⬤ | ⬤ | ⬤ |
| Gorodetskaya 2005 | ⬤ | ⬤ | ⬤ | ⬤ |
| sJardine 2017 | ⬤ | ⬤ | ⬤ | ⬤ |
| Jesky 2016 | ⬤ | ⬤ | ⬤ | ⬤ |
| Lee 2005 | ⬤ | ⬤ | ⬤ | ⬤ |
| Manns 2003 | ⬤ | ⬤ | ⬤ | ⬤ |
| Manns 2002 | ⬤ | ⬤ | ⬤ | ⬤ |
| Manns 2009 | ⬤ | ⬤ | ⬤ | ⬤ |
| Neri 2011 | ⬤ | ⬤ | ⬤ | ⬤ |
| Ortega 2013 | ⬤ | ⬤ | ⬤ | ⬤ |
| Ortega 2009 | ⬤ | ⬤ | ⬤ | ⬤ |
| Ortega 2007 | ⬤ | ⬤ | ⬤ | ⬤ |
| Pan 2018 | ⬤ | ⬤ | ⬤ | ⬤ |
| Wong 2019 | ⬤ | ⬤ | ⬤ | ⬤ |
| ⬤ low risk of bias/high quality; ⬤ unclear; ⬤ high risk of bias/low quality. | | | | |
